# Editorial: Visible Light-Responsive Photocatalysts for Energy Production

**DOI:** 10.3389/fchem.2019.00304

**Published:** 2019-05-07

**Authors:** Junguang Tao, Bing Li

**Affiliations:** ^1^School of Materials Science and Engineering, Hebei University of Technology, Tianjin, China; ^2^Institute of Materials Research and Engineering, Singapore, Singapore

**Keywords:** heterogeneous catalysis, metal-free photocatalysts, water splitting, oxygen evolution, hydrogen evolution, toxic pollutant removal

With the rapid growth of industry, there are growing concerns related to energy shortage, environmental pollution and global warming. Energy exhaustion, climate warming and environmental pollution are interrelated and mutually reinforcing. Searching for efficient and clean renewable energy has become a common topic for scientists all over the world. Among the many renewable energy sources, solar energy is a superior one due to its long life, unlimited and high power, pollution-free nature, on-site access and other advantages. However, suitable photocatalysts are usually needed to accelerate the reaction in photolysis of water or photodegradation of organic pollutants. In addition, the photocatalyst can be used in the areas of CO_2_ reduction for producing carbon neutral fuels without a net increase in atmospheric CO_2_ concentrations associated with fossil-derived alternatives (Izumi et al.). Because sunlight is made up of 47% visible light, it is appropriate to develop and utilize the visible light-responsive photocatalyst for the above fields. For water decomposition, when a semiconductor is used as photocatalyst, the bandgap should be >1.23 eV to meet the thermodynamic requirements. Meanwhile, the band gap of the semiconductor with visible light response should be <3.0 eV, because the broader band gap will lead to the lower utilization efficiency of visible light (>400 nm). Since the discovery that semiconductor titanium dioxide (TiO_2_) materials have the function of photocatalytic water decomposition (Asahi et al., 2001), this technology of using solar energy conversion to provide clean energy has been attracting much attention. The visible light photocatalysts can be classified into different categories, such as metal or metal-free compounds, organic or inorganic, homogeneous or heterogeneous photocatalysis, etc. They can also be categorized according to the substances they catalyze. All these materials have certain advantages in their respective applications. However, no material has performed well in all respects. The research object has extended from the initial inorganic to organic-inorganic hybrid materials, such as metal-organic skeleton and porous coordination polymers (Chen et al.). With continual development, the material system began to expand also from a single material to heterogeneous catalysis which could provide better charge separation efficiency for better photocatalytic performance due to the interfacial band alignment. For instance, the close contact and the match of bandgap structure between the heterojunctions of Ag_3_VO_4_ nanoparticles and Bi_2_O_2_CO_3_ micro-flowers boost the separation of electron-hole pairs, which accounts for the activity enhancement (Li et al.). Similarly, p-n heterojunctions of flower-like AgI/BiOCOOH also exhibit enhanced photocatalytic activities owing to the improved separation of charge carriers (Li et al.). The main active species responsible for the pollutant degradation were found to be the holes (h^+^) and superoxide radical (•O^2−^) (Li et al.). So far, the most effective photocatalysts are metal-based materials. However, the high cost and heavy metal toxicity of these photocatalysts limit their usage (Zhao et al.). For the metal-free photocatalysts, Wang et al. reviewed the recent advances in visible light-responsive boron nitride (BN)-based photocatalysts, including their synthesis, characterization and mechanism of activity enhancement. In addition, graphitic carbon nitride (g-C_3_N_4_), as an attractive metal-free photocatalyst, has drawn worldwide research interest recently due to its easy synthesis, earth-abundant nature, physicochemical stability and visible-light-responsive properties. Zhao et al. summed up the recent progress in designing and synthesizing two-dimensional (2D) g-C_3_N_4_ and g-C_3_N_4_-based nanocomposites (Zhao et al.). In addition, Zhao and coworkers discussed modifications and characterization of 2D g-C_3_N_4_ and g-C_3_N_4_ based cocatalysts, including their optical absorptions band structures and interfacial charge transfer behaviors. To better understand the system in theory, this article also summarizes the recent research based on density functional theory calculations. Qi et al. studied the photocatalytic performance of Ag-loaded g-C_3_N_4_ porous nanosheets. They found that loading of Ag NPs improved the response to the visible light for g-C_3_N_4_ and increased the production rate of photogenerated electron-hole pairs. On the other hand, porous organic polymers (POPs), known for their high specific surface area and abundant porosity, can be easily designed and constructed at the molecular level. POP provides a confined molecular space for the interaction of photons, excitons, electrons and holes, therefore featuring great potential in catalysis. The recent research progress of POP for photocatalytic water splitting was reviewed by Chen et al. The design principle, synthesis strategy and the relationship between structure and photocatalytic hydrogen and oxygen evolution performance were introduced. POPs have demonstrated their ability as versatile platforms for water splitting in all of the systems that have been intensely investigated. Photosynthesis exists widely in nature, relying on green plants and other plants, and collects solar energy through chloroplasts, converts CO_2_ and water into energy-rich organic compounds, and releases oxygen, which not only generates new energy but also effectively reduces greenhouse gas emissions. This process has inspired researchers to simulate photosynthesis for solar energy storage. The main purpose of artificial photosynthesis is to synthesize effective catalysts like chloroplasts. To achieve this goal, we need to better understand the working principle of the photocatalysts. Alongside this road, Izumil et al. found that photocatalytic conversion of CO_2_ into methane using Pd/TiO_2_ photocatalyst proceeds faster using water rather than hydrogen (Izumil et al.). The reason is attributed to the oxygen vacancy formation through the reaction of TiO_2_ with water.

The research of visible light-responsive photocatalysts for new energy production is a multidisciplinary research field, which needs a series of systematic engineering from material design, mechanism research and so on. This requires interrelated research strategies. The research progress of visible-light photocatalysts is very helpful for the supply of renewable energy and the treatment of the increasing environmental pollution. Although the research in this area is still in the progress stage, its goal is to solve the current and upcoming sustainable energy problems. The current research will provide new ideas, methods and feasible ways to solve these problems.

## Author Contributions

All authors listed have made a substantial, direct and intellectual contribution to the work, and approved it for publication.

### Conflict of Interest Statement

The authors declare that the research was conducted in the absence of any commercial or financial relationships that could be construed as a potential conflict of interest.
